# Remote photoacoustic sensing using speckle-analysis

**DOI:** 10.1038/s41598-018-38446-x

**Published:** 2019-01-31

**Authors:** Benjamin Lengenfelder, Fanuel Mehari, Martin Hohmann, Markus Heinlein, Erika Chelales, Maximilian J. Waldner, Florian Klämpfl, Zeev Zalevsky, Michael Schmidt

**Affiliations:** 1 Institute of Photonic Technologies (LPT), Friedrich-Alexander-Universität Erlangen-Nürnberg (FAU), Konrad-Zuse-Straße 3/5, 91052 Erlangen, Germany; 2 Erlangen Graduate School in Advanced Optical Technologies (SAOT), Paul-Gordan-Straße 6, 91052 Erlangen, Germany; 3 Tulane University, Biomedical Engineering, New Orleans, LA 70118 USA; 4 Department of Medicine 1, Friedrich-Alexander-Universität Erlangen-Nürnberg (FAU), Ulmenweg 18, 91054 Erlangen, Germany; 5 Faculty of Engineering, Bar-Ilan University, Ramat-Gan, 52900 Israel

## Abstract

Laser surgery is a rising surgical technique, which offers several advantages compared to the traditional scalpel. However, laser surgery lacks a contact-free feedback system which offers high imaging contrast to identify the tissue type ablated and also a high penetration depth. Photoacoustic imaging has the potential to fill this gap. Since photoacoustic detection is commonly contact based, a new non-interferometric detection technique based on speckle-analysis for remote detection is presented in this work. Phantom and *ex-vivo* experiments are carried out in transmission and reflection-mode for proof of concept. In summary, the potential of the remote speckle sensing technique for photoacoustic detection is demonstrated. In future, this technique might be applied for usage as a remote feedback system for laser surgery, which could help to broaden the applications of lasers as smart surgical tools.

## Introduction

Laser surgery enables accurate, fast and contact free tissue treatment^1–3^. Furthermore, it offers decreased bleeding and a higher patient acceptance rate compared to traditional surgical methods^4–7^. However, the main challenge of using lasers as surgical scalpels is the lack of information of the tissue type ablated at the surface and its depth extent. The lack of such information can lead to the iatrogenic damage of critical tissues that are meant to be preserved during the process. Normally, nerves or blood vessels which are below the tissue surface need to be preserved due to their important physiological function.

In this context, several tissue differentiation techniques have been investigated. In Laser-induced Breakdown Spectroscopy (LIBS) the optical emissions from the laser induced plasma during the ablation process are monitored. The atomic emissions from the created plasma plume are measured using a spectrometer. Further classification analysis provides detailed information on the tissue elemental composition allowing differentiation among various tissues^8^. Thus, it is possible to differentiate fat, muscle, nerve and skin tissue with very high sensitivities and specificities. Only the differentiation between fat and nerve tissue is challenging due to the fat-containing myelin sheath covering nerves. However, also in this case sensitivities higher than 95% are achieved^9^. In addition, cartilage and bone could be differentiated with a sensitivity and specificity of 100%^10^. LIBS achieves high differentiation accuracy. However, it only offers information of the tissue at the surface, the influence of contaminants is not yet analysed and *in-vivo* experiments have not yet been done.

A different approach for tissue differentiation is the detection of acoustic process emissions during laser surgery. By acquiring the acoustic signals during ER:YAG-laser osteotomy using a piezoelectric accelerometer a clear differentiation between cortical and cancellous bone can be achieved, which might help to guide laser osteotomy and to preserve essential tissue structures^11,12^. The acoustic transducers used for data acquisition are contact based. However, for clinical application, a remote, non-invasive differentiation setup is desired.

Optical techniques which analyse the optical properties of the tissue are remote and allow tissue differentiation. For Diffuse Reflectance Spectroscopy (DRS), the diffused reflection of a specific wavelength range is captured. Using statistical data analysis a clear differentiation between tissue structures can be achieved. Sensitivities of higher than 92% are achieved for the differentiation between skin, muscle, mucosa, fat and nerve tissue^13^. Furthermore, nerve, salivary gland and bone can be differentiated with sensitivities greater than 83%, which might improve oral and maxillofacial laser surgery^14^. DRS is also capable of distinguishing hard tissues: cortical bone, cancellous bone, cartilage and nerve^15^. DRS offers non-contact, non-invasive tissue differentiation. Nevertheless, spatial information on the region of interest is missing and also it might suffer from interference from the stray light in surgical environments.

Optical Coherence Tomography (OCT) offers spatial resolution and was also suggested for laser surgery guidance. OCT is capable of guiding the ablation location and imaging the dynamic changes of surgical laser ablation for brain, liver, kidney, lung and muscle^16^. Evaluation of tissue carbonization, thermal tissue damage and ablation crater can here be achieved. Furthermore, OCT was successfully tested as an automated feedback system for hard tissue surgery^17^. Ablation depth measurement and also classification of underlying tissue structures was achieved^18,19^. However, since OCT provides only a limited scattering contrast for soft tissue, its usability is limited for soft tissue differentiation. In addition, the penetration depth for OCT is limited to less than 2 mm for soft tissue which is not sufficient for some laser surgery applications.

The emerging imaging modality Photoacoustic Tomography (PAT) combines a high penetration depth for soft tissue with a high optical contrast^20^. As PAT is based on the absorption of light after short pulse excitation and the generation of acoustic waves in tissue, the absorption coefficient is the contrast origin. Since the absorption coefficient differs strongly for different tissue components high contrast values can be achieved. It is for example possible to image blood vessels clearly due to the high absorption of hemoglobin compared to other tissue chromophores in the visible and near infrared range. Since the scattering in soft tissue for sound is up to a factor of 1000 less than the scattering of light, this high imaging contrast is also combined with a high penetration depth of several millimetres^21^. This penetration depth is higher than for optical only methods and since the ablation depth per pulse in laser surgery is in the *μm*-range, it is sufficient for a laser surgery feedback system.

Contact based ultrasound transducers are the common technique for photoacoustic signal detection. These transducers offer limited tissue access with the need for mechanical contact and would limit the field of view in case of usage during a laser surgery which makes them not suited for a laser surgery feedback system. A non-contact, stable detection system would help to adapt PAT as a feedback system for laser surgery. One way would be to use air-coupled piezoelectric transducers or non-contact optical based approaches. However, these techniques also come with limitations for a laser surgery feedback system. Air-coupled piezoelectric detectors suffer from lack of sensitivity and are still too big for minimally-invasive medical applications^22^. In the non-contact beam deflection technique, the deflection of a probe beam over the object is monitored using a position sensitive detector^23,24^. When the photoacoustic signal reaches the surface, a small fraction is also transmitted to the surrounding air leading to pressure changes which deflect the probe beam. However, this modality is not appropriate for surgical usage since a probe beam needs to be scanned above the whole object of interest. Furthermore, the detection bandwidth is limited due to the probe beam size.

Interferometric setups offer non-contact sensing and a higher detection bandwidth. Here, the surface displacement after photoacoustic signal generation is monitored for the initial pressure reconstruction inside the object. A Mach-Zehnder interferometer was used to measure surface displacement of phantoms after photoacoustic excitation with a temporal resolution of 4 ns and a displacement sensitivity of 0.3 nm^25^. A resolution of smaller than 200 *μ*m and a penetration depth of 1 cm can be achieved. Non-contact photoacoustic imaging was also performed using a fiber based Mach-Zehnder interferometer^26^. In this case, imaging was done on tissue mimicking phantoms and chicken skin. A resolution down to 0.3 mm with a penetration depth of more than 1 cm was reached using a Fabry-Perot-interferometer^27^. Here, the surface deformations of a chicken breast and calf brain specimen were measured after photoacoustic excitation. All the mentioned interferometric detection principles need to scan the sample surface for image acquisition. Non-contact photoacoustic sensing can be done without spatial scanning by using full-field speckle interferometry. For this modality, the object deformations of an area on the surface are captured in one measurement^28^. Nevertheless, repeated photacoustic excitation is needed for time resolved signal acquisition. The mentioned interferometric approaches offer the advantage of high temporal and spatial resolution. Nevertheless, they are expensive and need a complicated setup which is noise sensitive. Right now, there is no easy, robust and remote detection modality for photoacoustic tomography which could be integrated in a surgical laser system. In this work, a new, easy and robust detection modality for remote photoacoustic sensing is presented, which could help to improve the current state of the art in laser surgeries if successfully implemented as a feedback system. Therefore, the remainder of this work will focus mainly on the potential of the technique for a laser surgery feedback system. However, it should be noted that this technique opens a new window towards detecting photoacoustic signal in general.

## Results

### Remote photoacoustic sensing in transmission-mode

#### Phantom Measurements

**Figure 1:**
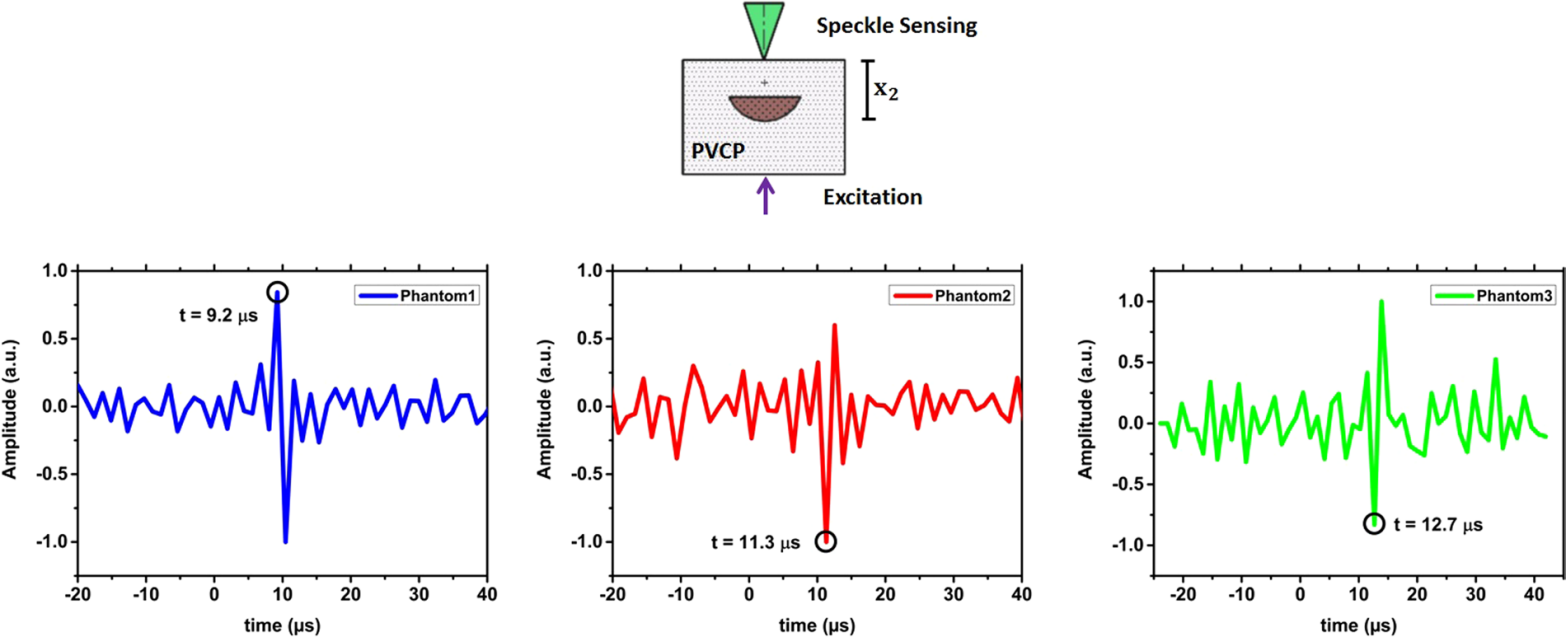
The temporal vibration profile of the phantom surfaces measured in transmission-mode is shown. Negative time points are related to measurements before the photoacoustic excitation. For the three phantoms, the initial peak which is generated by the photoacoustic signal is marked.

**Figure 2:**
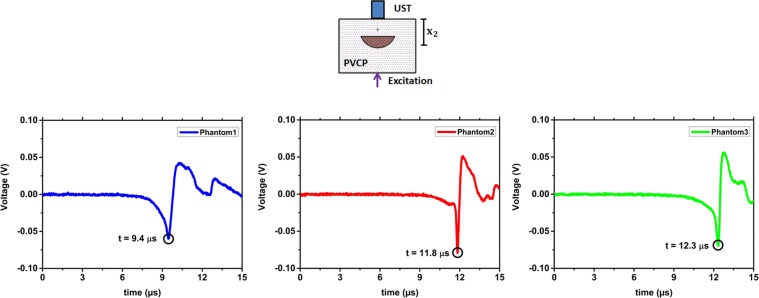
The phantom measurements in transmission-mode are verified using a contact ultrasound transducer (UST). For the three phantoms the first peak of the photoacoustic signal is marked which show the initial generated photoacoustic signal at the lower absorber surface.

The first experiments are performed on the optical phantoms made of PVCP (Polyvinylchloride plastisol). From phantom-1, phantom-2 and phantom-3 are respectively 56, 65 and 66 measurements analysed. The photoacustic measurements are done in transmission mode: photoacoustic excitation and remote detection take place on opposite phantom sides. Figure 1 shows measurement results for the three phantoms. The detection time of the first peak in the temporal vibration profile of the phantom surface is marked with a black circle. For each phantom, the detection time of the photoacoustic signal matches the geometrical distance of the absorbing target to the phantom surface (*x*_2_) considering the time interval between the video frames. For phantom 1 the initial peak is detected at 9.2 *μ*s. By using the speed of sound, a distance of 12.2 mm is calculated which matches *x*_2_ = 12.8 mm for phantom 1 (Fig. 1). For phantom 2 and phantom 3 the initial peak is detected at 11.3 *μ*s and 12.7 *μ*s, respectively. Distances of 15.1 mm and 16.9 mm can be calculated between the phantom surface and the absorber. This calculation is again in agreement to the phantom dimensions considering the uncertainty in the measurements. The SNR for the three phantom measurements is considerably high and decreases as expected with increasing *x*_2_: for phantom 1 (SNR = 10.1), phantom 2 (SNR = 6.3) and phantom 3 (SNR = 5.2) due to the lower deformation amplitude at the surface. Here, it needs to be noted that a penetration depth of 16.9 mm is high enough for the usage as a potential optical feedback system during laser surgery. For verification, the photoacoustic signal is also detected in transmission mode using a contact ultrasound transducer. Figure 2 shows the captured transducer signals. The time of the initial photoacoustic signal peak is marked. This time is related to the maximal pressure at the surface and consequently to a high surface tilt which can be detected by the speckle sensing technique. The times are in agreement to the physical dimensions of the phantoms. Slight differences can be explained with mechnical measuring errors during phantom preparation. In addition, the pressure peak times are also in agreement to the acquired detection times using remote speckle analysis considering the measurement uncertainty. The initial surface expansion after the photoacoustic excitation results in a positive pressure on the piezo element of the transducer element which is related to a negative voltage signal as seen in Fig. 2.

**Figure 3:**
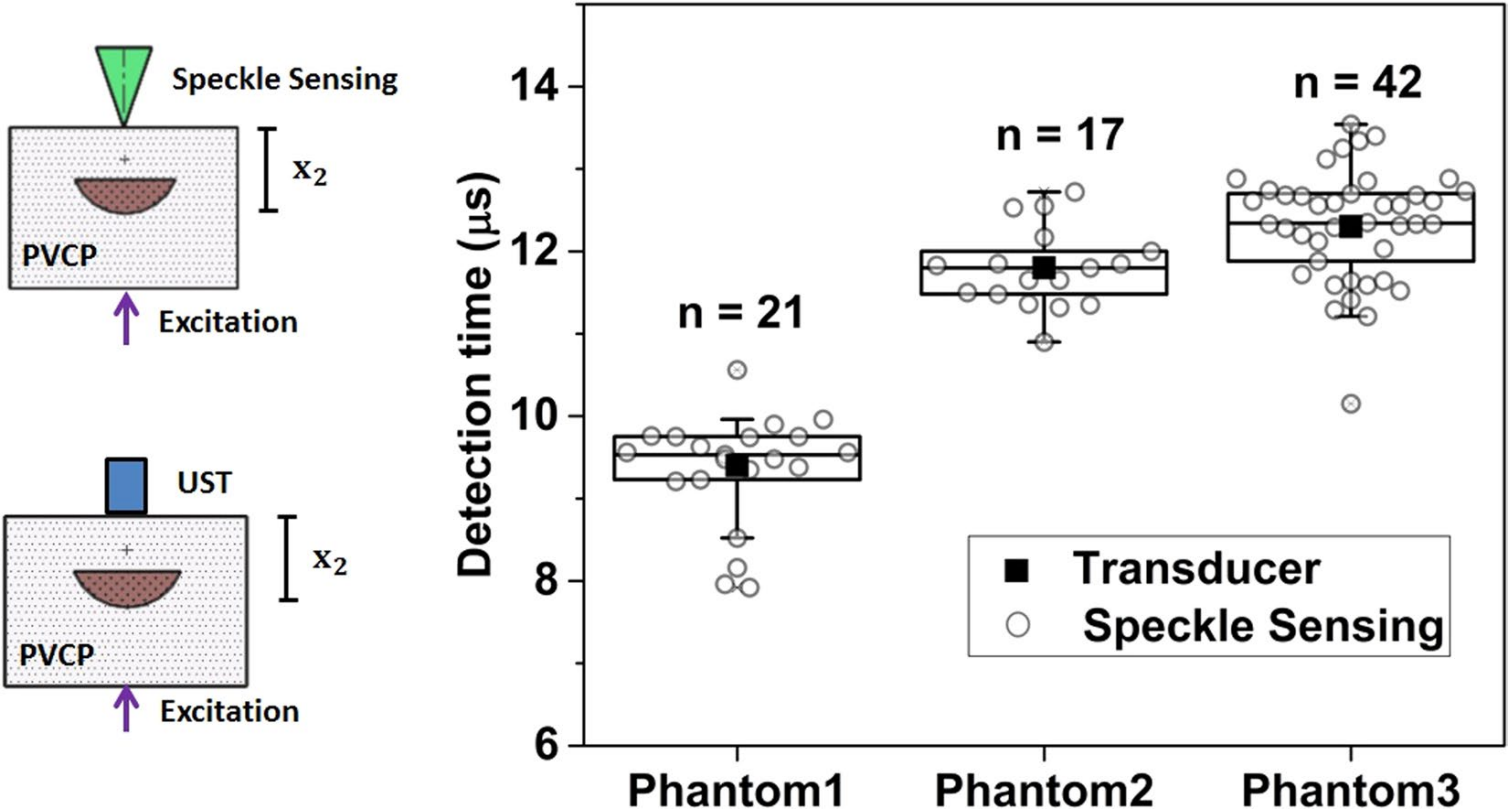
Box plot of the photoacoustic detection times using speckle sensing for the phantoms. The corresponding photoacoustic detection times using an ultrasound transducer are marked with an rectangle. This state of the art measurements match the time interval for speckle sensing.

Figure 3 shows the box plot of all detection times of the photoacoustic signal using speckle analysis and the times for the corresponding ultrasound transducer detection. It is clearly visible that the detection times of the transducer match the time intervals defined by the speckle sensing. Outliers can be explained by a slight misalignment of the cw-illumination spot or the short pulse excitation from the central phantom axis leading to an increased distance to the excited acoustic signal.

#### *Ex-vivo* Measurements

The *ex-vivo* sample is also analysed in transmission-mode by analysing 17 measurements with a SNR bigger than 4. The average detection time for the photoacoustic signal is 5.5 ± 1.0 *μ*s. Since the acoustic signal passes fat tissue and also PVCP, an average speed of sound of 1400$\frac{{\rm{m}}}{{\rm{s}}}$ can be assumed here. A value of 7.7 ± 1.4 mm for *x*_2_ can be reconstructed. This reconstruction matches the geometrical distance for *x*_2_ of 7.0 mm within the uncertainty. The transducer measurement shows the corresponding pressure peak at 5.2 *μ*s which matches the phantom geometrical dimensions and the speckle sensing data. The transducer data shows a second signal peak at 7.4 *μ*s which is related to a laser induced signal generated at the surface where the short laser pulse hits the tissue. This detection time matches the measured *ex-vivo* phantom height H of 10.4 mm using *c* = 1400 $\frac{{\rm{m}}}{{\rm{s}}}$.

**Figure 4:**
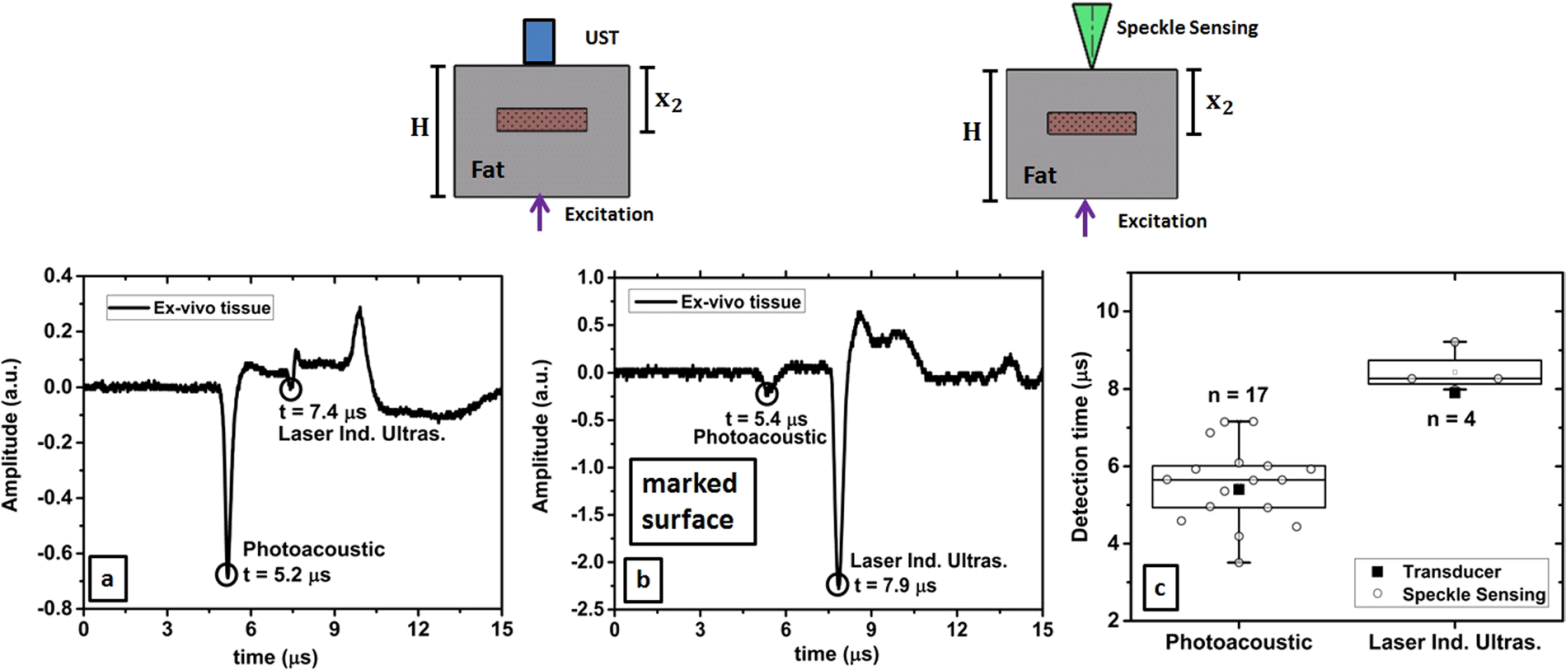
(**a**,**b**) The ultrasound transducer measurements in transmission-mode are shown with the corresponding detection times of the photoacoustic and laser induced ultrasound signal. (**b**) For the marked surface, the laser induced ultrasound signal is stronger than the photoacoustic signal. (**c**) Detection times using speckle sensing and its standard deviation for the photacoustic signal and for the laser induced ultrasound signal at the phantom surface. The state of the art measurement using the ultrasound transducer matches the time interval for speckle sensing.

A further experiment is done in order to exclude that the measured signal using speckle sensing is not the laser induced ultrasound generated at the phantom surface where the laser is coupled into the phantom. To do so, the surface opposite to the speckle sensing surface is marked using a black spray. This leads to a high absorption of the laser pulse directly at the surface and generates a strong acoustic signal here. In total four measurements are analysed and an average arrival time for the initial acoustic signal is measured at 8.4 ± 0.5 *μ*s using speckle sensing. This detection time matches the ultrasound detection time (7.9 *μ*s) and Fig. 4 shows the captured transducer signals for the unmarked and marked sample surfaces together with the box plots for the detection times of the photoacoustic signal using speckle analysis. For the marked and unmarked surface the photoacousic signal and laser induced ultrasound signal are detected at similar times. The slight differences can be explained with the deformation of the tissue during experimental handling. Furthermore, it is visible that the laser induced signal at the surface increases at the costs of the photoacoustic signal for the marked surface due to the high absorption of the spray. For both signal modalities, the transducer detection times match the time intervals defined by speckle analysis. For the laser induced ultrasound the transducer detection time is not that close to the median value for the speckle analysis measurements which can be explained by two reasons. First, there is again tissue deformation during the experiment. And second, due to the ablation of the black spray by the short laser pulse, it is not possible to carry out more measurements which might have improved the statistics.

### Remote photoacoustic sensing in reflection-mode

**Figure 5:**
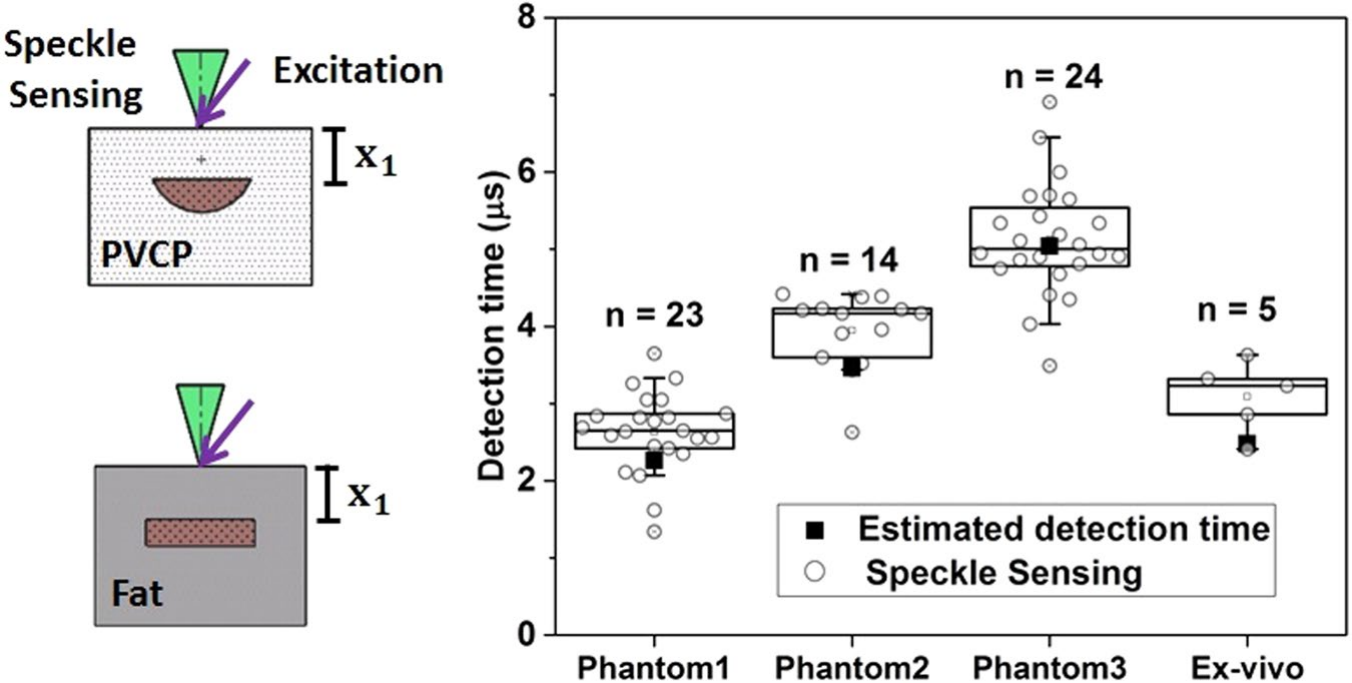
Detection times using speckle sensing and its standard deviation for the photoacoustic measurements. The theoretical arrival time of the photoacoustic signal matches the time interval for speckle sensing.

The optical phantoms and the *ex-vivo* sample are also measured in reflection-mode. From phantom-1, phantom-2, phantom-3 and the *ex-vivo* sample are respectively 42, 36, 52 and 10 measurements analysed. Figure 5 shows the box plot for the detection times of the photoacoustic signals with SNR bigger than 4 using the speckle analysis and the calculated detection time using *x*_1_ and the speed of sound. It is visible that the calculated detection times match the time intervals defined by the speckle sensing for the phantoms. However, there is the trend that the median value for the speckle sensing detection time is later than the estimated detection time. This can be explained by the fact that the estimated detection time is calculated using *x*_1_ which is the shortest distance between the sample surface and the absorber. In reality, light scattering leads to photoacoustic signal generation at locations at the absorber which have a bigger distance to the detection spot of the sample surface than *x*_1_. This leads to a later detection time using the speckle sensing technique. For the *ex-vivo* sample, the speckle sensing detection times are also too late. This can be explained by the mentioned reason and in addition the tissue may have been deformed during experimental handling.

## Discussion

### Selection of Sample Material and Assumptions

Selecting a proper phantom material for photoacoustic sensing is difficult. A perfect phantom should possess the following properties: tissue-like and controllable acoustic properties, tissue like and controllable optical parameters, values for relevant thermoelastic properties, realistic and versatile architecture, long-term stability, reproducibility of preparation^29^. There are many materials available which can be used for photoacoustic experiments. However, there is no standard phantom material for photoacoustics since none of the so far used materials fulfils all of the mentioned properties.

Polyester and resin have already been used in photoacoustics since they offer long term stability^30^. However, they do not mimic tissue mechanical properties due to their high speed of sound and acoustic attenuation. Water based materials like gelatin and agarose were also used for photoacoustic measurements^31,32^. However, they lack long term stability and degrade quickly over some days by loosing water. This makes them unsuitable for long term measurements hindering the reproducibility of measurements. Another material that has been widely applied in photoacoustics is polyvinyl alcohol (PVA)^33^. For this material, the acoustic and optical properties cannot be tuned independently of each other. In addition, its preparation process is long^34^. PVCP is a suspension of PVC in liquid plasticizer which undergoes mutual dissolution when heated^33^. This material has already been investigated as photoacoustic material and shows stability for at least six months. Furthermore, the preparation process is easy and takes approximately one hour. Realistic and versatile phantom architectures are also feasible since the heated, fluid PVCP is poured into a prepared mould and solidifies quickly. By the addition of softener, the speed of sound and acoustic attenuation can be adjusted to the targeted tissue type. The density of PVCP is similar to water and thus matches most soft tissue types^29^. Furthermore, the optical properties can be tuned to the aimed tissue type by adding scatterers and absorbers during the preparation process. Considering all the needed properties for photoacoustic phantoms, PVCP seems to offer the best properties and is selected as phantom material in this work.

For the demonstrated proof of concept of remote photoacoustic detection using speckle-analysis, the phantoms used in this work provide a strong optical absorption contrast which is not found in real tissue for an excitation wavelength of 1064 nm. However, the selected excitation wavelength offers reduced Rayleigh scattering which leads together with the high absorption contrast to a strong and clear photoacoustic signal and allows the approximate reconstruction of the initial pressure source after optical excitation using a single measurement of the surface vibrations by time of flight. Next to the strong optical contrast there is a second assumption: The acoustic properties of the absorber and the scattering matrix are matched since the same material is used. Consequently acoustic reflections at the boundary between absorber and surrounding can be neglected. However, this is an assumption which can be transferred to soft tissue whose mechanical properties are very similar.

The absorber size used in this work is not found in real tissue. However, this big absorber size is needed for the proof of concept study in this work since the frame rate of 823.500 Hz of the high-speed camera limits the photoacoustic imaging resolution. With this frame rate it is possible to resolve surface changes at a frequency of approximately 400.000 Hz which leads to a spatial resolution of *δx* = 3.4 mm using *c* = 1330 $\frac{{\rm{m}}}{{\rm{s}}}$. Consequently, an absorber height of *h* = 9 mm and an absorber diameter of 17 mm are used in this work in order to ensure an excitation of an absorber area bigger than *δx*. In addition, this big absorber structures also ensure a photoacoustic surface deformation and resulting tilt which is sufficient for the remote detection approach. These two factors enable the reliable detection of the photoacoustic surface displacements using speckle-analysis.

### Selection of Imaging System

The imaging system was selected according to the resulting speckle diameter *SD*, resolution and imaging speed. The speckle size in a distance *Z* to the illuminated surface (imaging plane) is dependent on the illumination diameter *D* and the wavelength. It can be calculated according to eq. (1)^35^.

1 $$SD=\frac{\lambda Z}{D}$$

For this work the following parameters are used: *D* = 300 *μ*m, *Z* = 2 cm and *λ* = 532 nm. Using eq. (1) leads to *SD* = 35 *μ*m. This size can be detected by several pixels using the imaging system in this work (Resolution of 2.8 *μm*). Since the resolution is enough to capture one speckle, it is possible to detect the induced speckle movements due to surface deformations.

The sampling rates for photoacoustic signal detection is normally several megahertz (*MHz*). However, there is not any camera available that can provide this high acquisition rate for remote photoacoustic detection using speckle sensing. The used camera in this work (Vision Research (USA), Phantom v1210) is one of the fastest high-speed cameras available and offers 823,500 frames per second at a resolution of 128 × 16 pixels. This sampling rate is high enough for the proof of concept of remote photoacoustic signal acquisition by speckle analysis. However, for precise signal acquisition, a faster optical detection systems is required in future.

The setup is also designed according to the minimal detectable surface tilt *α*_*min*_. The correlation algorithm is capable to detect shifts of $\frac{1}{20}$ of the camera pixel size. This value defines the minimal detectable shift for the secondary speckle pattern. Considering the magnification of the factor of 10 of the objective a minimal detectable shift of the primary speckle pattern (*x*_*p*_) of $\frac{1}{200}$ pixel size can be calculated. Using this value and eq. (2), *α*_*min*_ is calculated at 4 × 10^−4^ ° (*Z* = 2 cm, pixel size 28 *μ*m). Horstmann *et al*. measured a maximal photoacoustic surface displacement of 50 nm for a silicone phantom with a cubic absorber with a diameter of 1 mm and a radiant exposure of 32 $\frac{{\rm{mJ}}}{{{\rm{cm}}}^{2}}$^28^. For the study shown in this work even bigger absorber sizes and higher excitation exposures are used which should result in bigger deformation amplitudes than 50 nm with a surface tilt bigger than 4 × 10^−4^ °. Due to this comparison, the noise equivalent detection tilt (4 × 10^−4^ °) is considered as sufficient small for the detection of the photoacoustic signal in this work.

### Signal Quality and Behaviour

Based on the repeatability and on the successful verification of the transmission-mode results using a state of the art ultrasound transducer, it can be concluded that speckle sensing is a reliable technique for the temporal photoacoustic detection in transmission-mode on phantoms and *ex-vivo* tissue. It is also possible to reliably separate the photoacoustic signal from the laser induced surface signal using speckle sensing. Furthermore, it is demonstrated that speckle sensing reliably detects the photoacoustic signal in the reflection-mode. This is an essential step towards a feedback system for a smart laser scalpel. In this case, the excitation and remote sensing also need to be done from the same side.

**Figure 6:**
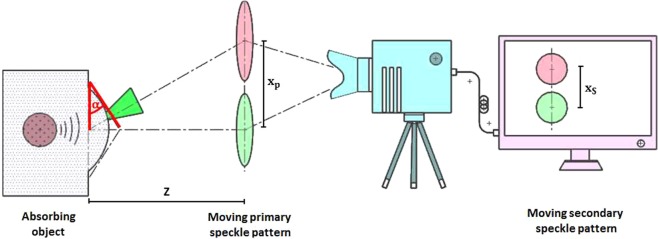
The generated photoacoustic signal of the absorbing object leads to a surface tilt *α*. This angle leads to a shift of the primary speckle pattern in the observation plane with distance *Z* to the object surface. By using a camera system, the secondary speckle pattern shift *x*_*s*_ can be calculated and it is possible to recover the surface tilt *α*.

For the remote speckle sensing technique, the movement direction of the primary speckle pattern and thus also the movement direction of the secondary speckle pattern depends on the direction of the surface tilt *α* (see Fig. 6 and eq. (2)). The correlation analysis of the secondary speckle video extracts the shift of the secondary speckle pattern (*x*_*s*_) related to the image center. A different direction for *α* results in a different movement of the secondary speckle pattern related to the image center and thus in a different sign for the temporal vibration profile. The sign for the initially detected *α* after photoacoustic excitation depends on the sensing location on the object surface. This effect explains the different signal behaviour in Fig. 1. The first signal peak for phantom 1 is positive, whereas it is negative for phantom 2 and phantom 3. A slight different displacement of the illumination beam from the phantom center may have taken place between these measurements which results in a different sign for the initial detected *α* for the same photoacoustic deformation.

Not all measurements carried out for this work show a clear signal peak. This can be explained by the low sampling rate for photoacoustic signal acquisition, the low photoacoustic surface amplitude and potential displacement of the cw-illumination. As already mentioned, the camera sampling rate is much too low for photoacoustic detection and thus the signal might not be resolved completely. Based on the results from Horstmann *et al*., it can be assumed that the maximal photoacoustic surface deformation in this work is in lower *nm*–range at the phantom centre^28^. This low amplitude decreases quickly at points which are off the phantom centre due to acoustic attenuation. As a consequence, a displacement of the illumination beam from the phantom center influences the signal quality of the temporal vibration profile acquired by speckle sensing.

Furthermore, it needs to be mentioned that there are two main advantages compared to contact transducers, which influence the signal quality. First, there is no frequency dependent sensitivity for speckle sensing compared to transducers, which show a resonant behaviour at a specific frequency. This advantage together with the development of faster imaging systems might lead to a very clear signal acquisition in future. Second, the size of the area on the surface, where the vibration detection takes place can be scaled easily by focusing or by changing the imaging aperture. Consequently, the spatial sampling of the surface can be very fine compared to bulky transducers. This fact together with a fine scanning approach might lead to a clear signal reconstruction for speckle sensing.

### Conclusion and Outlook

This work reports on a new all optic non-interferometric system for detecting photoacoustic signals remotely based on speckle sensing. A proof of concept is performed by photoacoustic measurements in transmission-mode on optical phantoms mimicking optical and mechanical properties of soft tissue. These measurements are verified using a contact ultrasound transducer which is state of the art for photoacoustic signal detection. Furthermore, the feasibility of remote photoacoustic detection on biological tissue is demonstrated using *ex-vivo* fat tissue. The potential for a successful application as a feedback system for laser interventions is also highlighted with the measurements in reflection-mode on optical phantoms and *ex-vivo* fat tissue. A penetration depth of up to 6.6 mm was achieved which is high enough for a potential laser surgery feedback system.

For future work, it is necessary to reduce the cw-illumination irradiance for speckle generation and the pulse energy for photoacoustic excitation, since exposures which are above the allowed limits are used. This can be achieved with a more sensitive detector unit which allows the usage of low power cw-lasers. Remote vibration sensing using the speckle analysis has already been successfully demonstrated on biological tissue using low power cw-lasers^36–38^. The more sensitive detector system will also allow remote photoacoustic detection on absorbing surfaces in contrast to the only-scattering surfaces like PVCP and fat used in this work. The SNR and penetration depth of the remote speckle sensing technique needs to be evaluated for lower excitation pulse energies. In addition, the sensitivity of the remote speckle sensing technique should be determined. It is possible to measure the surface deformations with a vibrometer in order to specify the minimal detectable tilt using speckle sensing.

Precise image reconstruction would not be possible with the used setup due to the used sampling rate of 823500 Hz and due to the fixed cw-illumination. Consequently a faster detector unit based on diodes together with a flexible, scannable illumination needs to be developed. By scanning the cw-illumination over the field of interest on the object, point by point signal acquisition could be performed with repeated photoacoustic excitation. For each measurement point, the speckle pattern is captured and temporally tracked after photoacoustic excitation with the diode array and the temporal oscillation profile is computed. After data acquisition, it would be possible to reconstruct the initial pressure distribution of the excited volume using back projection. Assuming an acquisition rate of 30 MHz, a reconstruction precision in tissue below 100 *μ*m could be reached. This is considered to be accurate enough for a potential remote laser surgery feedback system. Considering a common repetition rate of 10 Hz for the removal of skin cancer using an Er:YAG or CO_2_ laser it is realistic to measure at 50 × 50 spots with an acquisition duration of 4 *μ*s each^39^. This acquisition time results in a penetration depth of approximately 5 mm in tissue which would be sufficient for the usage as a potential laser feedback system. Tissue differentiation would then be possible, since the absorption characteristics and thus the photoacoustic signal strength and the reconstructed initial pressure value change between tissue types. For cancerous skin tissue and for blood vessels the absorption coefficient increases due to an increased hemoglobin concentration^40^. Also, the absorption properties for the skin constituents epidermis, dermis, subcutaneous fat differ strongly^41^.

This future work packages might lead to more realistic experiments and later to *in-vivo* trials for laser surgery. If successfully implemented in a laser surgery system, the speckle-sensing technique might provide non-contact feedback with a high optical contrast and penetration depth. The technique might for example assist the laser removal of skin cancer like basal cell carcinoma or squamous cell carcinoma since contact-free photoacoustic imaging of the region of interest could be done between the ablation pulses in order to measure the remaining ablation thickness and to prevent healthy tissue. Furthermore, it also holds the potential to be implemented in an endoscopic setup since the speckle image can also be transferred using imaging fibres which would allow endoscopic remote photoacoustic sensing. This is interesting for endoscopic photoacoustic imaging and again for the usage as a laser surgery feedback system for endoscopic laser applications. In general, it could help to broaden the application of the laser for medical surgeries.

## Methods

### Signal Generation and Speckle-Analysis

In photoacoustic sensing, the absorption of a short laser pulse generates heat and leads to a thermo-elastic expansion which generates an acoustic signal^42^. This acoustic signal reaches the surface and deforms it. The deformation of the surface generates also a tilting with the angle *α* respective to the original undistorted surface. Figure 6 shows on the left side the generated surface tilt after photoacoustic excitation. The right side of Fig. 6 illustrates the speckle sensing technique. It is possible to detect this surface tilt with the speckle sensing technique. If a rough surface is illuminated with a cw-laser, a speckle pattern will be generated. This speckle pattern moves depending on the surface movements of the illuminated area. The surface movement and consequently the speckle pattern movement can be grouped in three degrees of freedoms: axial, transversal and tilting. If the observation plane distance for the primary speckle pattern fulfils the far field approximation, the speckle pattern movement is only dependent on the surface tilt and on the distance *Z* of the observation plane to the surface^43^. Equation (2) describes the resulting shift *x*_*p*_ of the primary speckle pattern in the observation plane with the distance *Z* to the tilted object surface. The shift *x*_*s*_ of the resulting secondary speckle pattern is defined by the magnification *M* of the optics and can be captured by the camera: ${x}_{s}=M{x}_{p}$. By calculating the cross-correlation between the video frames, the temporal shift of the secondary speckle pattern is calculated and the temporal surface tilt is recovered. Using the speed of sound *c*, it is possible to reconstruct the location of the initial pressure source in the object volume relative to the laser illuminated area.

2 $${x}_{p}=\,{\tan }(\alpha )Z$$

### Sample Preparation

**Figure 7:**
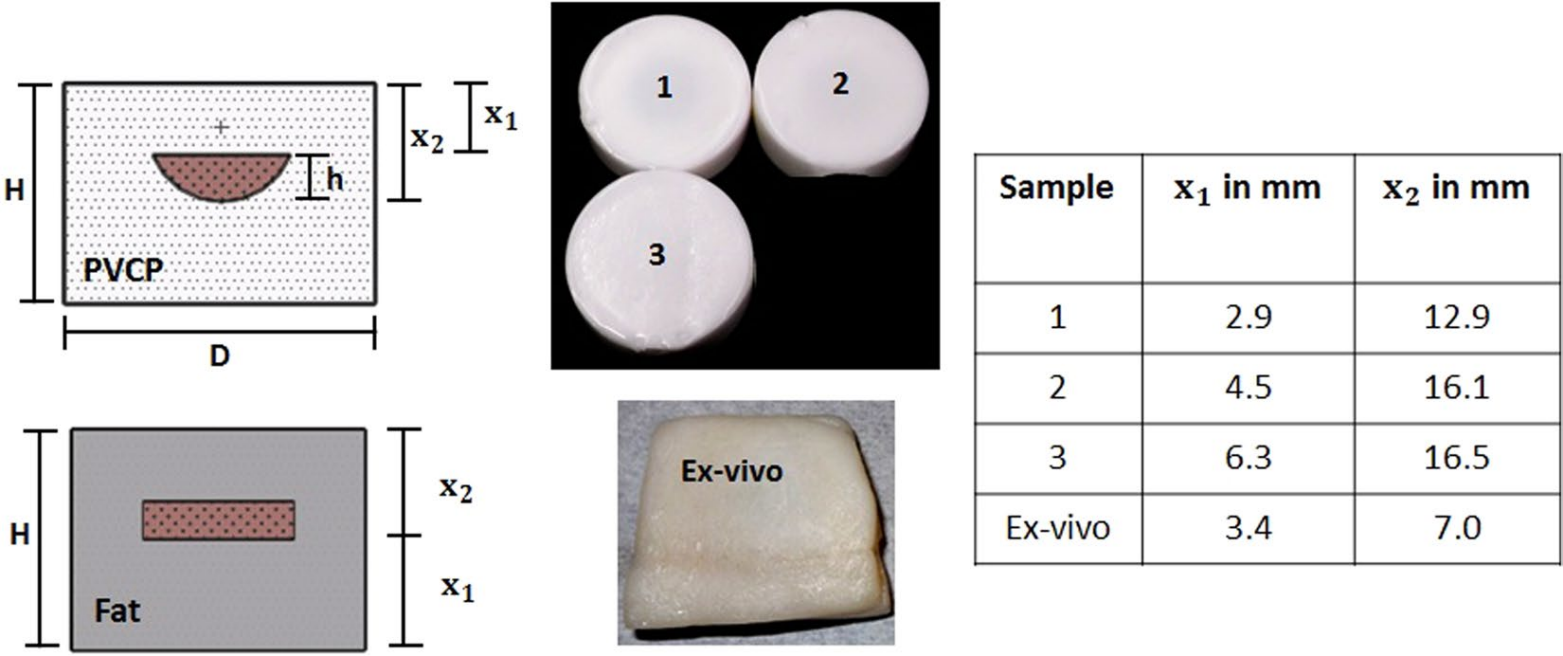
The PVCP phantoms used in this work consist of an absorbing hemisphere with a surrounding scattering matrix. The distance *x*_1_ between the hemisphere and the phantom surface is varied. The *ex-vivo* sample consists of an absorbing cylinder made of PVCP surrounded with fat tissue.

Figure 7 shows details of the experimental samples that include a tissue phantom and an *ex-vivo* porcine tissue obtained from a local supermarket.

For this work, Polyvinylchloride plastisol (PVCP, Standard Lure flex (medium), Lure Factors, Great Britain) is used as phantom material. The speed of sound *c* in the phantoms is measured using an ultrasound thickness measurement device (Mini Test 430, Elektro Physik, Germany) connected to a piezoelectric sensor head with a resonance frequency at 2 MHz at 1330 ms. The density $\rho$ is measured by volume displacement of ethanol at 1040 $\frac{{\rm{kg}}}{{{\rm{m}}}^{3}}$. The resulting acoustic impedance ($Z=\rho c$) of the used phantoms in this work is $1.38\times {10}^{6}\,\frac{kg}{{m}^{2}s}$ which is in good agreement with the values of soft tissue: The impedance of fat tissue is $1.4\times {10}^{6}\,\frac{kg}{{m}^{2}s}$ and for muscle $1.62\times {10}^{6}\,\frac{kg}{{m}^{2}s}$^44^.

In order to adjust the optical properties, additives are added during the preparation process. A black plastic color is added to change the absorption coefficient *μ*_*a*_ and *TiO*_2_-particles are added to adjust the reduced scattering coefficient ${\mu ^{\prime} }_{s}$. In this work, a color-concentration of 7 Vol-% and a *TiO*_2_-concentration of 4 $\frac{{\rm{mg}}}{{\rm{ml}}({\rm{PVCP}})}$ is used for the absorbing and scattering phantom parts. The optical properties for these concentrations were determined at the excitation wavelength 1064 nm using spectrophotometric measurements and Inverse Adding Doubling. In this work, the absorption coefficient for the absorbing phantom part is 1061 cm and the reduced scattering coefficient for the scattering part is 21 $\frac{{\rm{1}}}{{\rm{cm}}}$. The scattering coefficient for the absorbing part and the absorbing coefficient for the scattering part can be neglected.

For the experiments carried out in this work, three cylindrical PVCP-phantoms with a diameter of 46 mm are manufactured consisting of a scattering matrix and an absorbing core. The phantoms are produced in a three-step process. First, the bottom layer is manufactured and second, the absorbing core is produced using a hemispherical shape with a diameter *d* of 17 mm and a height *h* of 9 mm. Third, the absorbing target is put on the bottom layer and the cast is filled until the final phantom height *H* is reached. The geometrical distances are measured using a caliper. The distance *x*_1_ of the absorbing core to the phantom surface is varied for the first three phantoms. The *ex-vivo* sample consists of porcine fat. The tissue sample was prepared in a two-step process. First, two square-slices with a side length 35 mm are cut out of the fat. Second, a hole with the dimensions of the cylindrical target is cut out from one of the slices. Third, an absorbing target (PVCP) is pressed into the hole. Ultrasound gel is used to assure good acoustic coupling with the tissue. Finally, the two layers are pressed together. The speed of sound for the fat tissue is assumed at 1450 $\frac{{\rm{m}}}{{\rm{s}}}$^45^.

### Optical and Experimental Setup

**Figure 8:**
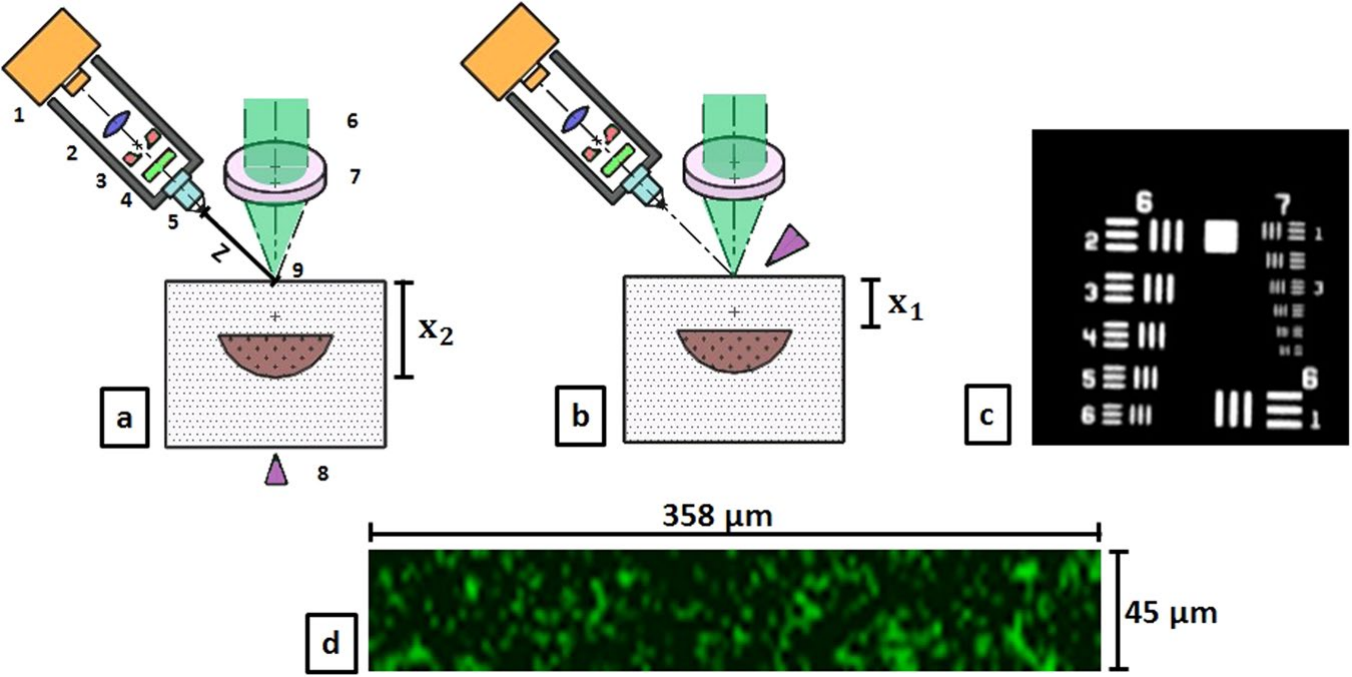
(**a**) Optical Setup for remote photoacoustic sensing in transmission-mode using speckle analysis. The detection unit consists of a high speed camera (1), lens (2), aperture (3), bandpass-filter (4) and microscope objective (5). The speckle pattern is generated by cw-laser beam (6) which is focused on the phantom surface (9) using a lens (7). The sample is excited with a short laser pulse aiming at the phantom centre (8) which triggers the high speed camera. (**b**) Optical Setup for remote photoacoustic sensing in reflection-mode. Excitation and sensing take place on the same object side. (**c**) The resolution of the imaging system was measured at 2.76 *μ*m (Group 7, Element 4) using a USAF 1951 Test Target. (**d**) Example of a speckle image captured with the imaging setup (128 × 16 pixel). The scales are added using the resolution, pixel size and the optical magnification $M=10$.

Figure 8 illustrates the optical setup used for remote photoacoustic sensing in transmission-mode (excitation and detection on opposite surfaces) and reflection-mode (excitation and detection on same surface) using speckle analysis. Photoacoustic excitation of the phantom is done using a single short laser pulse (Q-Smart 450, Quantel laser, Les Ulis (France)) targeting the phantom centre. The used laser has a wavelength of 1064 nm, a pulse duration of 5 ns and a beam diameter of 7 mm. For proof of concept a pulse energy of 40 mJ is selected for the phantom experiments in reflection-mode, leading to an energy exposure of 104 $\frac{{\rm{mJ}}}{{{\rm{cm}}}^{2}}$. For the transmission-mode measurements, a pulse energy of 50 mJ is used. The resulting energy exposure of 130 $\frac{{\rm{mJ}}}{{{\rm{cm}}}^{2}}$. Both exposure are higher than the maximum permissible exposure for single pulse photoacoustic excitation on soft tissue at 1064 nm (100 $\frac{{\rm{mJ}}}{{{\rm{cm}}}^{2}}$^46^). The high excitation energy generates a high photoacoustic signal amplitude which can clearly be detected at the phantom surface and helps to understand the physical principles of the new detection approach for proof of concept. A cw-laser (532 nm, 100 mW) illuminates the detection side of the sample surface for speckle generation and a high-speed video camera (Phantom v1210, pixel size 28 *μ*m, Vision Research, USA), which is triggered by the single short laser pulse and then detects the speckle pattern. The illuminated diameter on the sample surface is approximately 300 *μ*m. This results in a high exposure which is above the maximum allowed value for soft tissue. However, this exposure is selected in order to create a bright speckle pattern which can be detected by the optical imaging system for the demonstrated proof of-concept study. The optical system consists of an infinity corrected microscope objective ($NA=0.28$, working distance 34 mm), a bandpass for 532 nm, a mechanical aperture (diameter adjustable from 0.8 mm to 12 mm), a biconcave lens ($f=200\,mm$) and the sensor of the mentioned high speed camera. The aperture diameter was adapted for each measurement in order to achieve a high speckle contrast and sufficient sampling of the speckle size. The resolution of the optical system is measured about 2.8 *μ*m measured by a microscope test target (1951 USAF test target, Fig. 8). The distance of the imaging plane to the sample surface (*Z*) is 2 cm. After photoacoustic excitation, a video is captured with a sampling rate of 823,500 frames per second and a resolution of 128 × 16 pixels. This sampling rate is too low for precise photoacoustic sensing. However it is high enough for the proof of concept demonstration in this work. The sampling rate leads to a time window of 1.2 *μ*s between the frames. The captured video is analysed with Matlab R2015b (The MathWorks, Inc., Narick, MA, USA). By calculating the correlation between the video frames, the temporal vibration profile of the sample surface can be acquired. The time points of this vibration profile represent the time difference between trigger income and the end of an exposure interval of the high speed camera. For comparison, the temporal vibrational profile is normalized to the maximum and the signal to noise ratio $(SNR=\frac{1}{\sigma })$ is calculated using the standard deviation of the temporal vibration profile *σ* before short pulse excitation. For this work photoacoustic measurements in transmission-mode and reflection-mode, using the phantoms and the *ex-vivo* sample, are carried out. For the phantom measurement, more than 36 single measurements per phantom were carried out and analysed in order to ensure statistical relevance. For the photoacoustic *ex-vivo* measurements more than 10 measurements per sample were carried out. The reduced amount of measurements for the *ex-vivo* samples avoids the influence of tissue deformation or drying during the experiment. From these measurements, the measurements with a SNR bigger than 4 are analysed since they show a clear signal peak and box-plots are prepared.

Furthermore, for verification of the remote photoacoustic measurements, a broadband contact ultrasound transducer V111-RM, Olympus Corporation, Japan) with a resonance frequency of 10 MHz is used. Contact transducers are at the moment the state of the art for photoacoustic signal detection and this modality is considered s precise compared to the remote speckle sensing approach.

### Statistical analysis

All photoacoustic experiments were carried out at least 10 times and the illustrated box plots show the results of at least 4 measurements after the statistical analysis.
